# Supplementary material for: Salicylic Acid and Jasmonic Acid Increase the Polysaccharide Production of Nostoc flagelliforme via the Regulation of the Intracellular NO Level
Source: Foods. 2023 Feb 21;12(5):915. doi: 10.3390/foods12050915 (PMC10001311; doi:10.3390/foods12050915)
Supplement: Supplementary file 1 [file foods-12-00915-s001.zip › foods-2169752-supplementary.pdf]

Supplementary materials

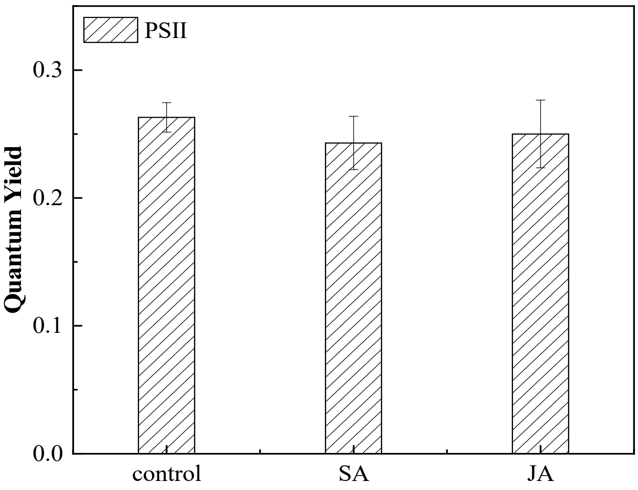

Figure S1. Effects of SA and JA on the photosynthetic system II electron yield of *N. flagelliforme*.

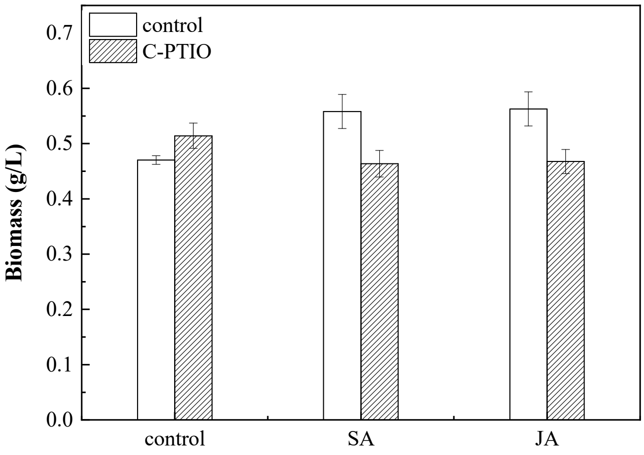

Figure S2. Effects of C-PTIO on the biomass of *N. flagelliforme*.

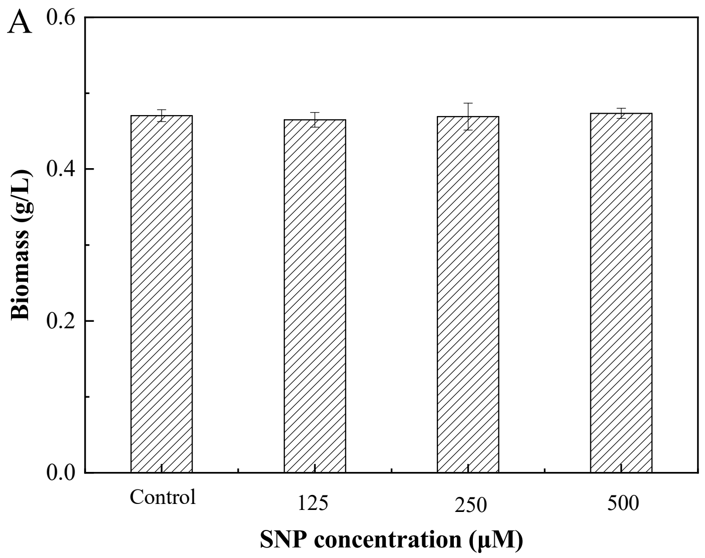

Figure S3. Effects of SNP on the biomass of *N. flagelliforme*.
